# Conceptual fluency in inductive reasoning

**DOI:** 10.1371/journal.pone.0225050

**Published:** 2019-11-21

**Authors:** Michael Dantlgraber, Tim Kuhlmann, Ulf-Dietrich Reips

**Affiliations:** Department of Psychology, University of Konstanz, Konstanz, Germany; University of Melbourne, AUSTRALIA

## Abstract

Psychological effects connected with fluent processing are called *fluency effects*. In a sample of 403 participants we test whether *conceptual fluency* effects can be found in the context of *inductive reasoning*, a context that has not been investigated before. As a conceptual manipulation we vary the use of symbols (persons and crosses) in reasoning tasks. These symbols were chosen to provide hints for the solution of the implemented tasks and thus manipulate fluency. We found evidence that these hints influence *ease of processing*. The proportion of solved tasks increased by 11% on average in the condition with conceptual hints, F(1,399) = 13.47, partial η^2^ = .033, *p* < .001. However, we did not find an effect of the conceptual manipulation on the *temporal perception* of the task. In a second study (*n* = 62) we strengthened our findings by investigating solution strategies for the tasks in more detail, 79% of the participants described the tasks in a way they were intended. Our results illustrate the advantages of the separation of *ease of processing*, *fluency experience*, and *judgments*.

## Introduction

**Fig 1:**
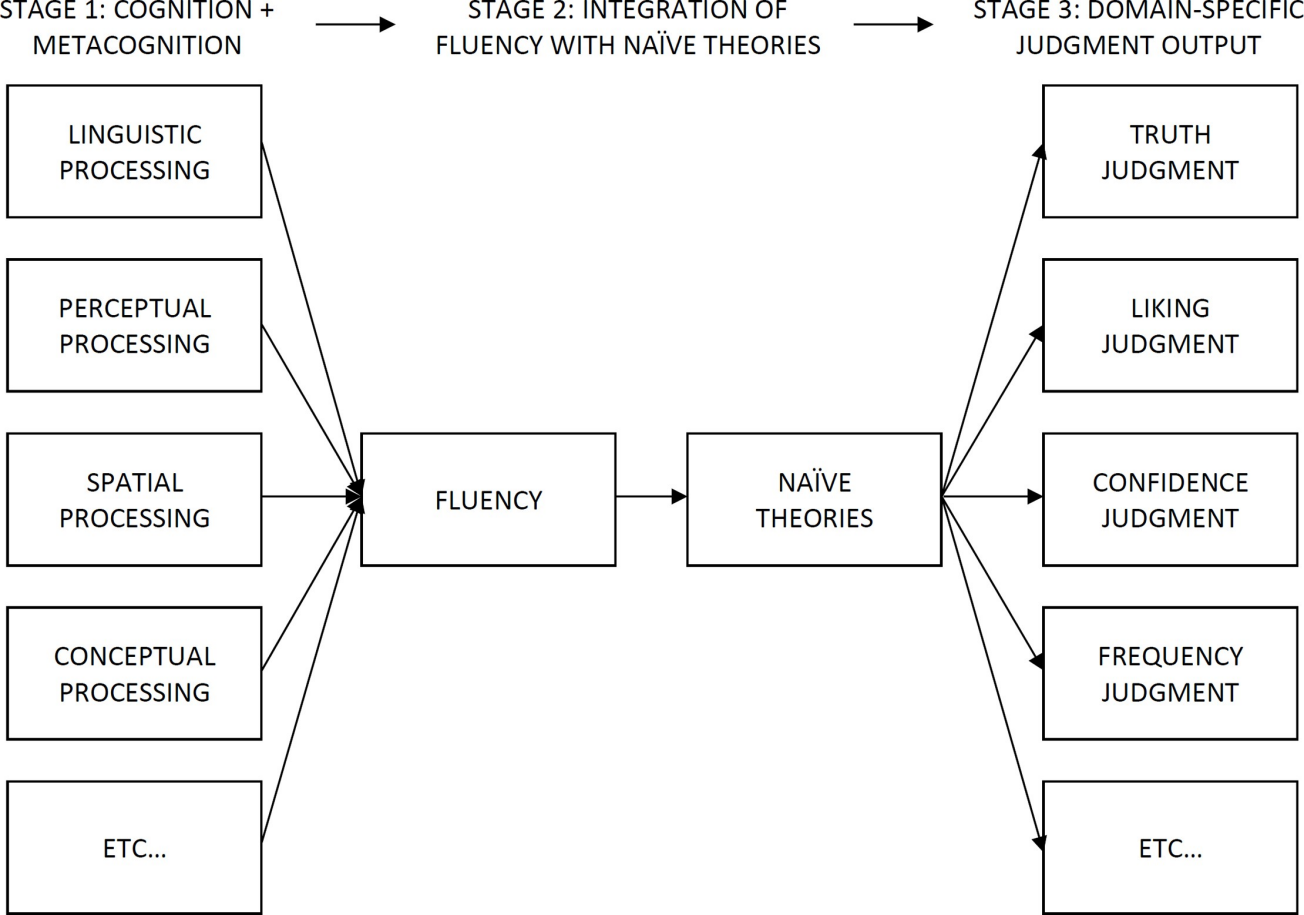
Model of assumed generalizability of fluency effects [4].

Fluency research has a long tradition in psychology. Initially, the term *fluency* was used when investigating effects of metacognition and cognitive monitoring [1] on judgments. In 1991 Schwarz et al. [2] established the *availability heuristic* [3] as an important fluency concept. It encompasses factors that influence memory retrieval and the effects of easy or difficult memory retrieval on judgments. Schwarz et al. [2] showed that participants’ evaluation of their own retrieval achievement can influence their judgments more strongly than their objective retrieval achievement. In 2009 Alter and Oppenheimer [4] reviewed fluency studies and demonstrated that “fluency is a ubiquitous metacognitive cue that accompanies cognition across the full spectrum of cognitive processes” (p. 232). Therefore, they propose an overall model that includes both the generation of fluency experience and naïve theories [5] that are traditionally used to explain participants’ judgments (Fig 1). The model assumes that irrespective of the determinant of fluency the subjective fluent or disfluent experience is comparable. The subjective experience then leads to a domain-specific output in the respective judgement domain. This allows for the transfer of fluency effects across domains.

However, even if the model is based on several findings the assumed generalizability of found effects to other fluency domains has not yet sufficiently been investigated. The present study thus aims to test whether the known effects of *conceptual fluency* can be generalized to *inductive reasoning* that has never been investigated in the past.

Note that Alter and Oppenheimer’s model [4] shown in Fig 1 does not represent the current state of research anymore. In this model the term *fluency* is equivalent with subjectively defined *processing fluency*, that is, the subjective experience of ease that ranges from fluent to disfluent [4]. Relatively objective measures of fluent processing like the number of recalled behaviors [2] are missing here. Such behavioral measures have been summarized under the label of *ease of processing* [6].

**Fig 2:**
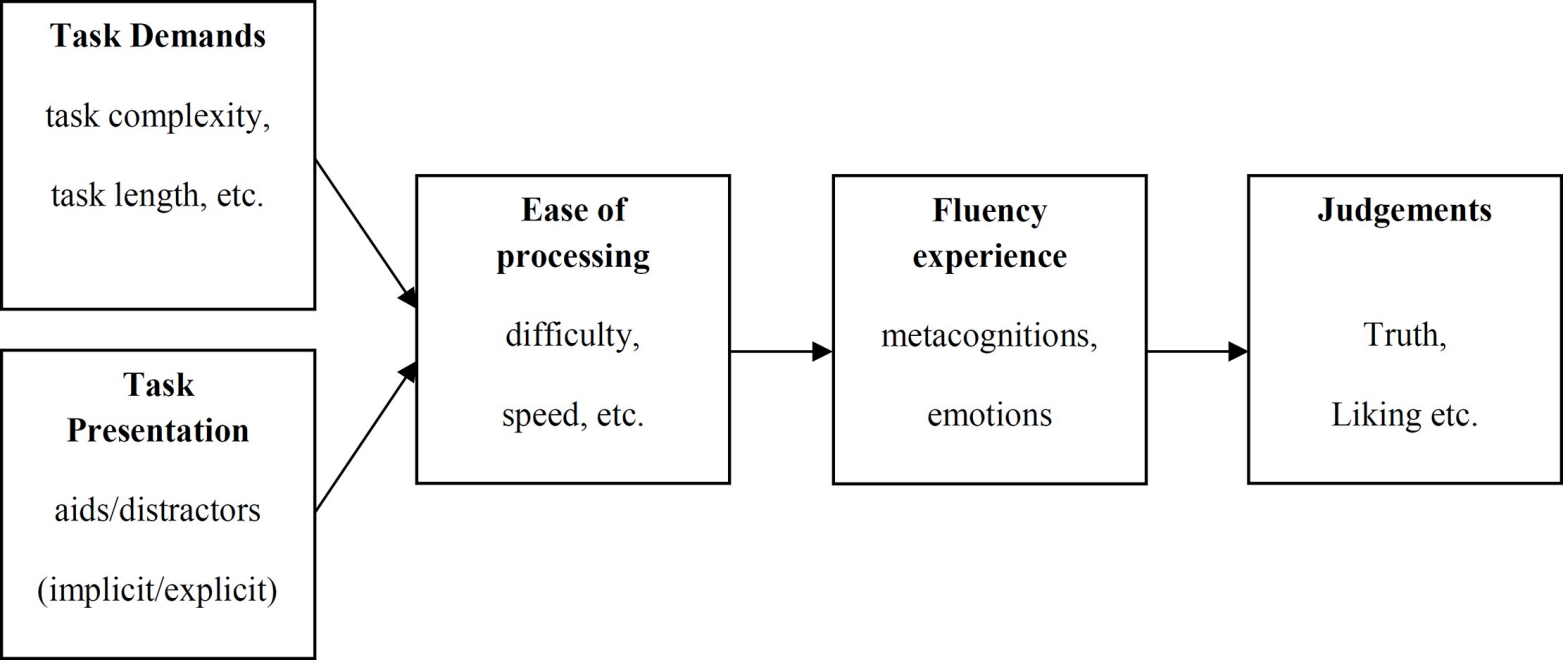
Important parameters that can be explicitly or implicitly varied in a fluency study.

Alter and Oppenheimer’s model shown in Fig 1 mainly emphasizes the assumed generalizability of known effects. Fig 2 summarizes the current state of research and shows which kind of parameters can potentially be measured or varied in a fluency study. This model helps us to better classify the tasks, measures and manipulations that are important to the present study in the area of inductive reasoning.

The differentiation between task demands and task presentation in the first stage is especially important when combining reasoning and fluency research. The last three stages of Fig 2 (*ease of processing*, *fluency experience*, *judgments*) represent further stages of processing and judgements in fluency studies. In reasoning research ease of processing (e.g. depending on the item difficulty) is typically varied by varying the complexity of the tasks. In contrast, in fluency research the ease of processing is typically varied by varying the presentation of the same tasks. Because we aim primarily at uncovering processes of fluency, we also use the same tasks in all conditions and only vary the task presentation. We use different symbols (persons and crosses; see *Methods section*) that contain more or less conceptual hints for the solution of the inductive reasoning tasks.

### Conceptual fluency

There is much evidence that processing can be facilitated by conceptual hints. The term *conceptual fluency* initially referred to several studies in which semantically related concepts are used. Reder [7] demonstrated that presenting words that are conceptually connected with the correct response have a positive effect on the rate of correct responses. For example, presenting the words “golf” and “par” facilitates answering the following question: “What term in golf refers to a score of 1 under par on a particular hole?” (correct answer: “birdie”). Because the presented words also appear in the question, results imply a facilitated memory retrieval.

Whittlesa [8] showed that words can be named more quickly when they are presented in a predictive semantic context. This is assumed to be caused by faster processing in this condition. Winkielman et al. [9] also underline the semantic aspect of conceptual fluency by defining it as “the ease of high-level operations concerned primarily with categorization and processing of a stimulus’ relation to semantic knowledge structures” (p. 200). In that regard they mention context congruity as an example of conceptual fluency. The present study aims to manipulate context congruity in inductive reasoning tasks to investigate effects of different task presentations on ease of processing (Fig 2). Further findings imply that a conceptual manipulation influences *temporal perception* [10], but does not influence *task duration* [11,12,13]. It seems that fluent processing leads participants to think that they have processed tasks faster, but this perception is not reflected in the actual task duration. For this reason, we included measurements of temporal perception and task duration in our study.

### Inductive reasoning

McGrew [14,15] introduced the term *fluid reasoning*, because *fluid intelligence* [16] cannot be differentiated from the ability that is called *reasoning* [17,18]. The term *fluid reasoning* describes the ability to adjust to new problems without needing to refer to acquired knowledge. McGrew called *inductive reasoning* and *deductive reasoning* the hallmarks of fluid reasoning. Inductive reasoning, which we measure with our tasks, mostly describes the ability to recognize connections and to derive and apply superordinate rules [18]. Note that in part of the literature the term *inductive reasoning* is also used to describe specific tasks where participants have to correctly generalize property attributions [19].

### The general inductive reasoning task type

**Fig 3:**
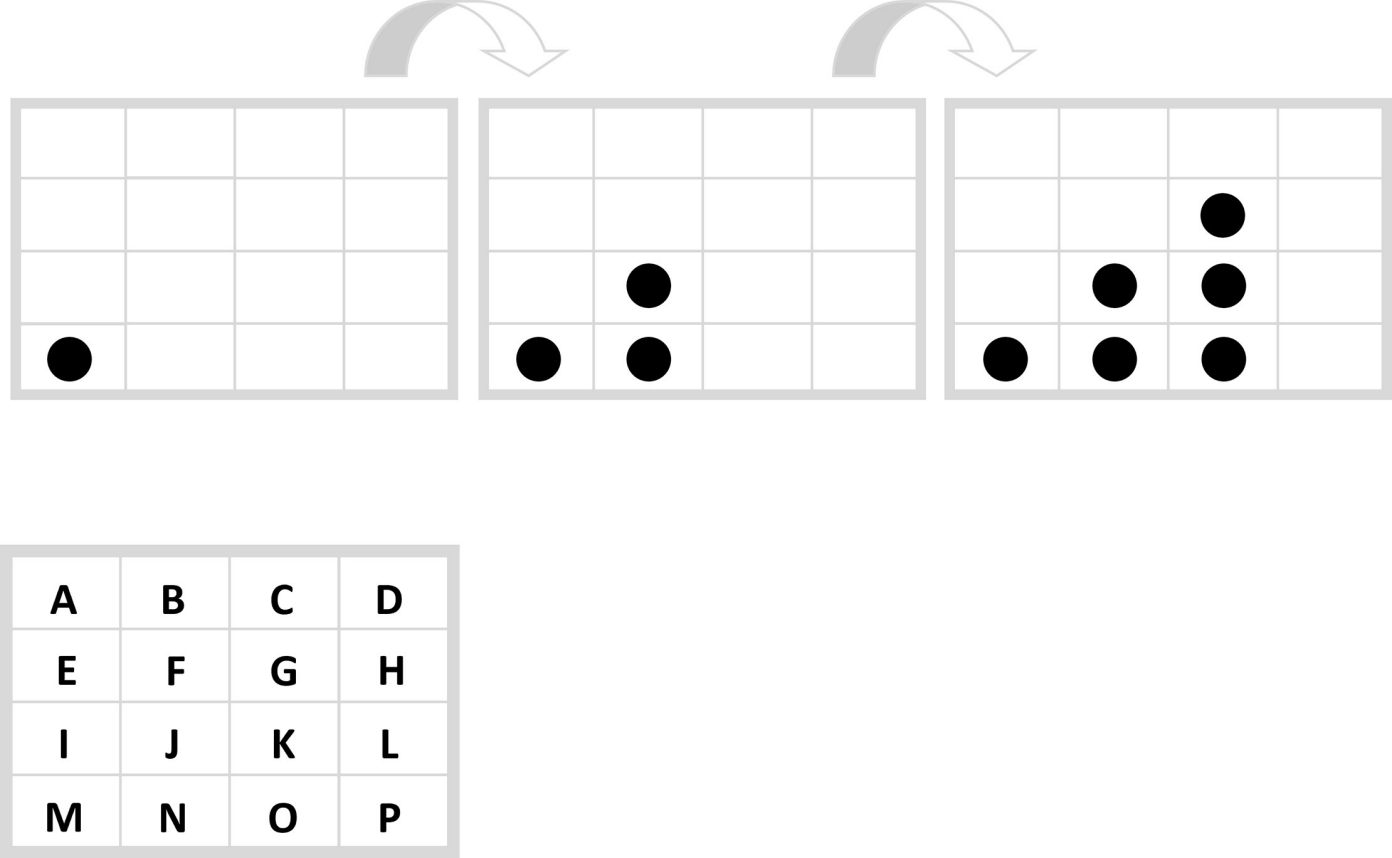
Inductive task example. “On which fields do four points appear in the next step?” Correct response: “DHLP”.

In order to unite reasoning and fluency research we developed a General Inductive Reasoning Task Type that offers the opportunity to more flexibly vary single tasks than usual. The most frequently used task types to measure inductive reasoning are matrices like the Raven Progressive Matrices [20] and numerical series. The General Inductive Reasoning Task Type is simply the combination of both, i.e. a series of matrices. Fig 3 shows an example of a General Inductive Reasoning Task Type. This task was also used as example in the instructions of the present study.

In a General Inductive Reasoning Task Type participants are asked to predict a matrix that was not yet presented. In order to find the right solution participants have to find the underlying rule that explains the changes from one matrix to the next. This matches the definition of inductive task types described above. In the example in Fig 2 four points are expected to appear in the fields “DHLP”. We used the General Inductive Reasoning Task Type to generate two tasks, the Filling Task and the Movement Task (see *Methods section*). These two tasks represent two different underlying rules to explain changes within the matrices: A filling rule that interprets the used symbols as a firm framework, and a movement rule that interpret the symbols as moveable. Each task can be presented in two conditions by either using symbols that are conceptually congruent with the task solution (i.e. they offer a conceptual hint to filling or moving) or by using symbols that are less conceptually congruent with the task solution. The tasks were created in a balanced way, so that each of the symbols we used (crosses and persons) can represent both the conceptually congruent and the less conceptually congruent condition.

### Hypotheses

According to the findings described above (see *Conceptual Fluency section*) we predicted the following effects regarding measures of objective ease of processing (rate of correct responses) and subjective judgments (temporal perception).

H1: Referring to Alter and Oppenheimer’s [4] assumed generalizability of fluency effects, we hypothesize that the relationship between a conceptual manipulation and the rate of correct responses [7] can be generalized to the context of inductive reasoning. We hypothesize that participants in the conceptually congruent condition solve more inductive tasks than participants in the less conceptually congruent condition.

H2: Further, we hypothesize that the relationship between a conceptual manipulation and temporal perception [10] can be generalized to the context of inductive reasoning. We hypothesize that participants in the conceptually congruent condition think that they have solved the tasks faster than participants in the less conceptually congruent condition.

## Methods

### Ethics statement

The data presented in this article were collected in full accordance with the the World Medical Association’s Declaration of Helsinki [21] and the Ethical Guidelines of the German Association of Psychologists (DGPs). Neither these guidelines nor German laws earmark formal ethic approvals when participants work on reasoning tests. For this reason we did not request formal ethics approval as is common at German universities when conducting these kind of studies.

The participation was completely voluntary. Moreover, participants were informed about the kind of tasks they had to expect. We did not obtain names or any other identifying information from the participants. Therefore the data we present in this article are completely anonymous.

### Study 1

The present study aims to test whether conceptual fluency effects can be found in the context of inductive reasoning.

#### Conceptual manipulation

**Fig 4:**
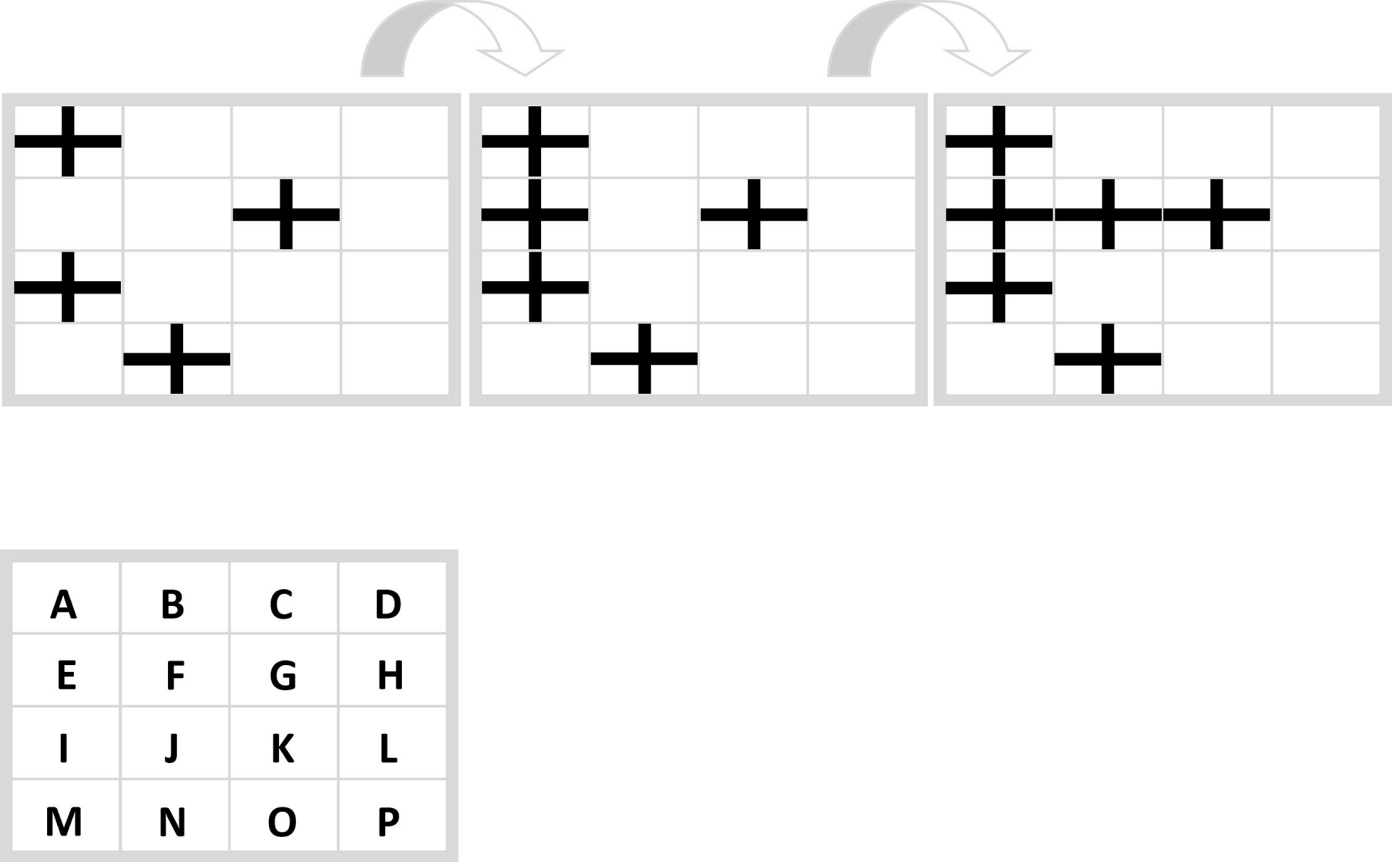
Filling task. “In which field does the next cross appear?” Correct response: “J”.

In order to conceptually manipulate *ease of processing* we developed two tasks, the Filling Task and the Movement Task. Fig 4 shows the Filling Task (“In which field does the next cross appear?”). There the following rule applies: An empty field, which is either vertically or horizontally enclosed by crosses, is to be filled with a cross. Therefore “J” is the correct response. (*Because the field at the bottom left of the matrices is not be filled in the first two steps*, *such a constellation of symbols is not covered by the underlying rule*).

**Fig 5:**
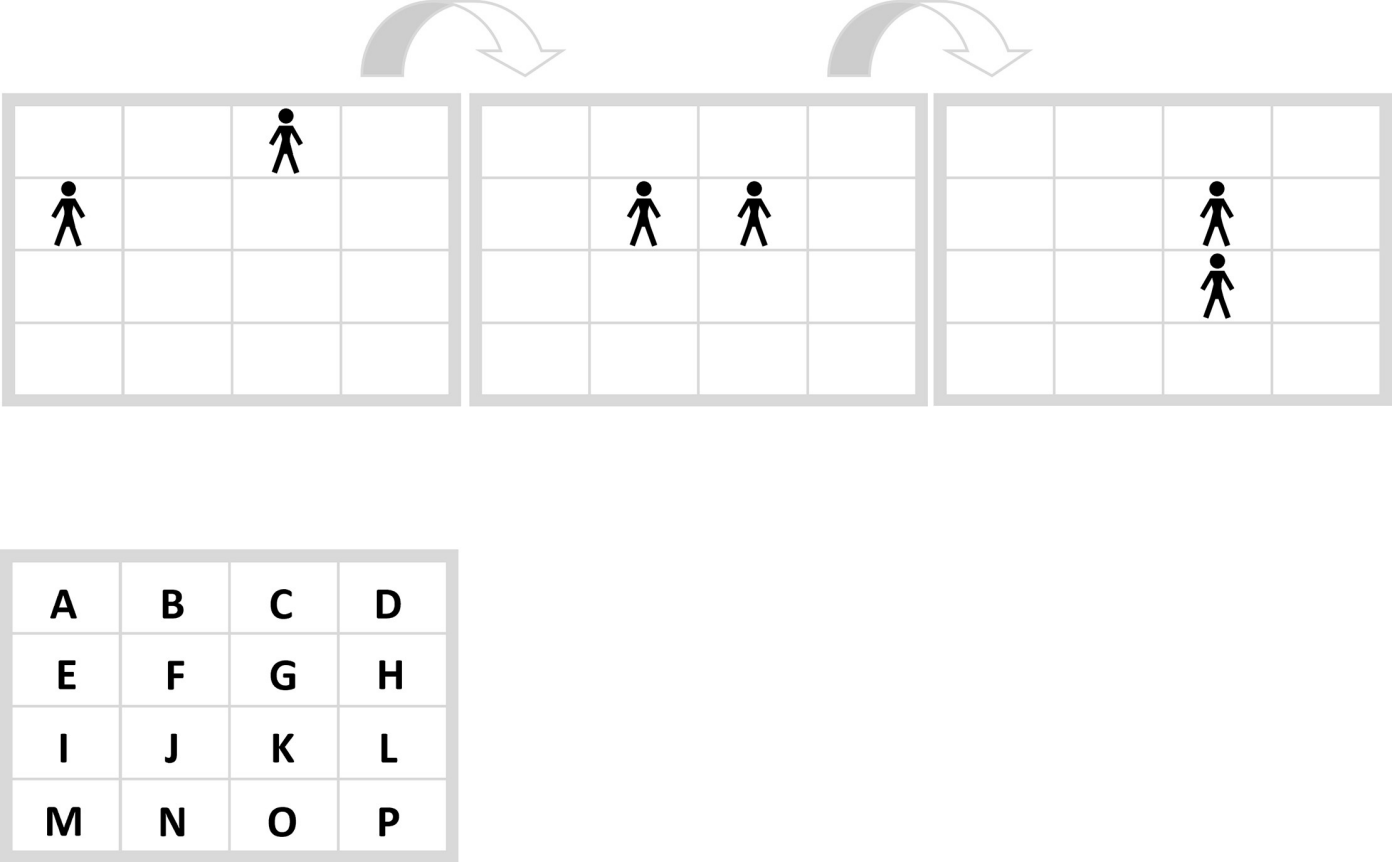
Movement task. “In which fields are two persons in the following picture?” Correct response: “HO”.

Fig 5 shows the Movement Task (“In which fields are two persons in the following picture?”). There the following rule applies: The person on the left side continuously walks from the left to the right side and the upper person continuously walks from the top downwards. Therefore “HO” is the correct response.

Both tasks, the Filling Task and the Movement Task have in common that the cognitive process that is required for task solution, that is, connecting horizontal or vertical lines (Filling Task) and moving objects (Movement Task), is assumed to be conceptually related to the implemented symbols. In everyday life participants are regularly confronted with persons that move (e.g. on a street) and with crosses that connect to form patterns (e.g. on a carpet). This relation may facilitate memory retrieval and therefore increases ease of processing. These tasks form the conceptually congruent condition.

**Fig 6:**
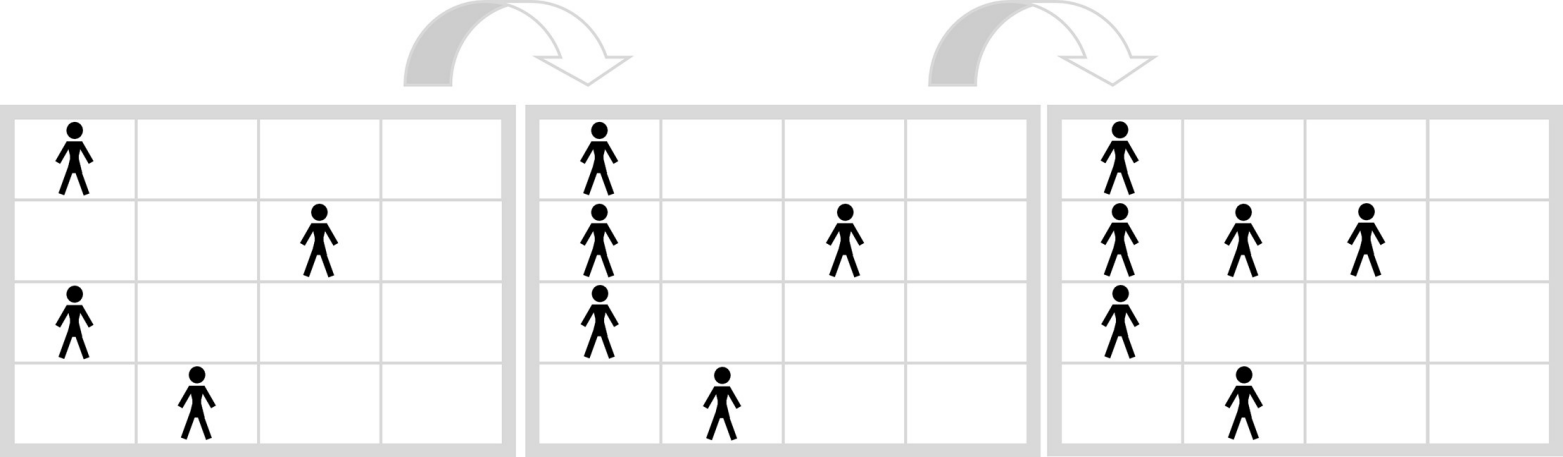
Filling task with persons (less conceptually congruent condition).

**Fig 7:**
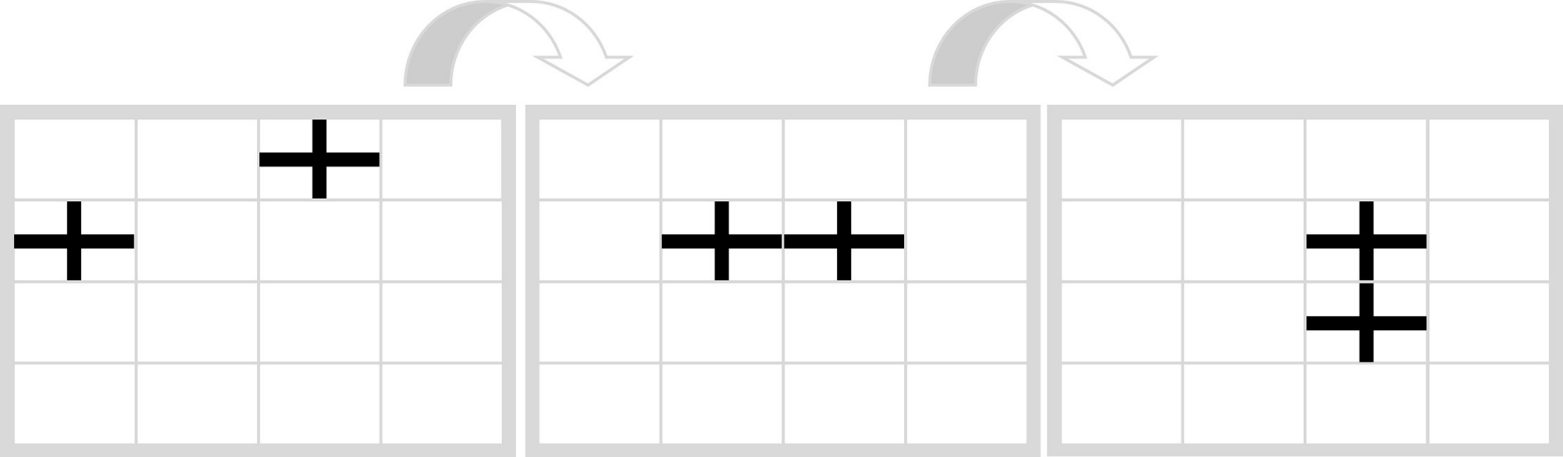
Movement task with crosses (less conceptually congruent condition).

We also developed the same two tasks with interchanged symbols, that is with *connecting persons* and *moving crosses* (Figs 6 and 7). These tasks form the less conceptually congruent condition, because in everyday life participants are comparatively less often confronted with crosses that move and with persons that connect to form patterns. In short, the symbols in this condition provide a weaker hint to the solution (Figs 6 and 7)

**Fig 8:**
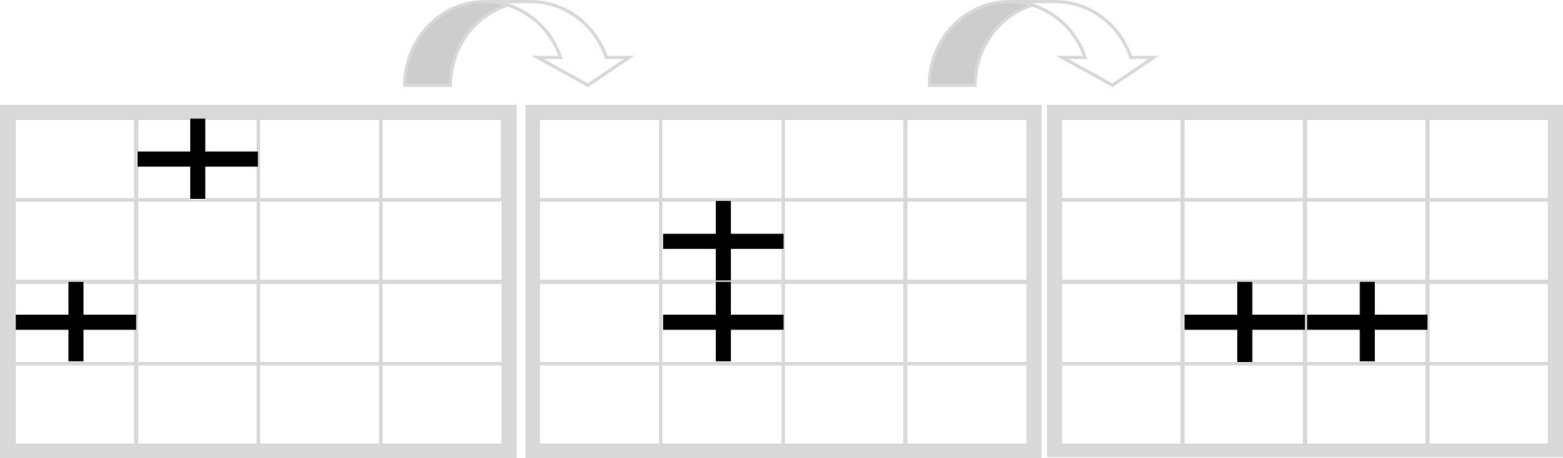
Parallel version of movement task with crosses (less conceptually congruent condition).

To increase the reliability of the measurements and calculate a continuous score, we developed a parallel version for each of the four tasks. In the parallel version of each task, rows and columns are switched in comparison to the original task. This keeps the logic and appearance of the task identical, but changes the correct response. The parallel task to the Movement Task with crosses (Fig 7) ist shown in Fig 8.

#### Additional manipulations

In the next step we developed a vertically mirrored version for each of the described eight tasks (Filling Task/Moving Task; congruent/incongruent; original/parallel). Mirrored tasks were developed to manipulate the direction of the task process and thus to control for a potentially confounding factor. It is possible that the General Inductive Reasoning Task Type (i.e. the series of the matrices) could activate a left-right orientation [22,23,24], which potentially has an influence on ease of processing. Mirrored versions of the tasks allow us to check for this possibility.

After each task participants gave a dimensional rating of their temporal perception, i.e. answered to the item “you have just engaged in solving a riddle. Please estimate how much time has passed until you clicked "next"” ranging from “very little time” to “very much time”. We varied the response format for this assessment using a seven-point Likert scale or a visual analogue scale [25,26,27]. Based on our previous research on scale types we thought that the continuous response options on visual analogue scales would be optimal for the assessment of time perception. However, in psychological research Likert-type scales are used more often. Therefore, we added both to check for potential methodological effects. The three dichotomous manipulations result in a 2 (*conceptual congruence*: strong, weak*)* x 2 (*task direction*: left-right, right-left*)* x 2 (*response format*: Likert, VAS) factorial design.

#### Procedure and measures

The web-based experiment was implemented via WEXTOR [28] (http://wextor.org:8080/Sp1R3niess/swa17fin/index.html?so=ms). Participants were recruited by student assistants who were instructed to each recruit at least 15 participants by using e-mail contacts or social media On the first screen (welcome page) participants gave informed consent and were asked to participate on a laptop or desktop computer via the Web to guarantee comparable procedures. This screen also included an adaptation of the Seriousness Check [29,30,26]. Participants were asked, if they would like to seriously participate on a laptop/PC or if they would like to look at the pages only on any device. On the second screen participants were asked to indicate their sex (male, female, other, no answer) and their age. On the next screen all participants were given the same task example illustrating the General Inductive Reasoning Task Type (Fig 3).

After this screen participants were randomly assigned to one of the eight groups of the 2x2x2 experimental design. Participants were asked to solve the original and then the parallel version of the Filling Task, followed by the original and then the parallel version of the Movement Task. In line with the typical scoring of inductive reasoning tasks, a correct response was scored as 1 and an incorrect response or a missing response was scored as 0. Depending on the experimental group the tasks were presented in their conceptually congruent or less conceptually congruent form (*conceptual congruence*), and in their left-right or right-left form respectively (*task direction*). After each of these four tasks participants were asked to estimate the amount of time it had taken them to complete the task on a seven-point Likert scale or a visual analogue scale (*response format*). The objective amount of time people spent on each task was measured in milliseconds within the web browser [31]. Each task was announced on a separate screen so that the subjective and the objective time measures had a clear starting point. The endpoint of the objective measure was the submission of a response via the “next”-button. Finally, after completing all these tasks, participants rated different short scales on coping humour, malevolence, nutrition, environmental concern, and information seeking tendencies. These ratings are unrelated to the study at hand.

#### Sample Study 1

In total, 1,555 participants clicked on the recruitment link and saw the first page. In 1057 cases, participants didn’t give a positive response to the Seriousness Check on the first page and thus their data were not included with the analyses. Also data were not included from participants who did not continue after arriving at the Seriousness Check page and data from scripts (“bots”) by search engines that catalogue the web. Data from 87 participants (5.6%) were not included that dropped out of the study before having finished the fourth task. Data from three participants (0.2%) were not included because they indicated an age below 18. Data from one participant (0.1%) was not included who probably paused the study while working on the third task having a study time of more than 5 hours. Data from two participants (0.1%) were not included because their mean task durations could be identified as clear outliers (more than three standard deviations away from the mean). They probably paused the study for a shorter time period. Finally, data from two participants (0.1%) were not included because their data were not recorded correctly.

In the final sample (*n* = 403) the participants’ reported age ranged from 18 to 84 years (*M* = 33.2, *SD* = 15.5). Nine of them (2%) did not state their age. The final sample reportedly consisted of 239 female participants (59.3%), 156 (38.7%) male participants, and two “other” participants (0.5%). Four (1%) participants gave “no response” and two (0.5%) did not respond without explicitly stating it.

#### Analysis

In the Sample 1 we chose analytical procedures that could consistently be used for all hypotheses. (1) We calculated ANOVAs using scores or means as dependent variables. (2) We retested effects with nonparametric tests to ensure robustness against violations of conditions for parametric tests.

#### Results

Regarding Sample 1 an ANOVA was calculated first. The number of correctly solved tasks (0–4) was used as the dependent variable. *Conceptual congruence* and *task direction* were used as factors. The factor *conceptual congruence*, F(1,399) = 13.47, partial η^2^ = .033, *p* < .001, showed a significant main effect supporting H1 that the conceptually congruent tasks were solved more often *(M* = 2.33, *SD* = 1.21) than the less conceptually congruent tasks *(M* = 1.87, *SD* = 1.29). *Task direction*, F(1,399) = 0.43, partial η^2^ = .001, *p* = .514 and the interaction of both independent variables F(1,399) = 0.53, partial η^2^ < .001, *p* = .820 did not reach significance.

**Fig 9:**
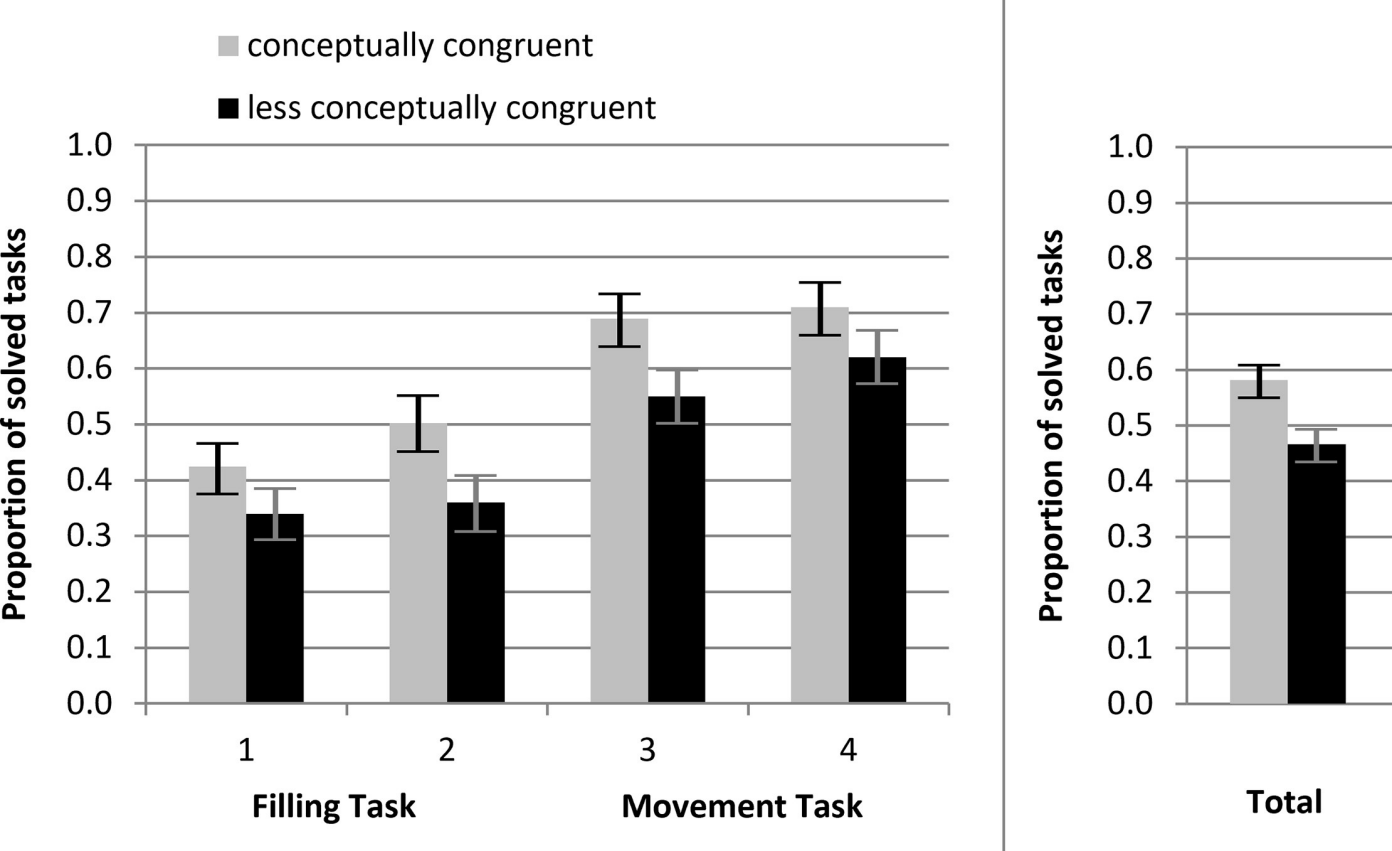
Proportions of solved tasks for the conceptually congruent and the less conceptually congruent condition. Task 1 = Filling Task, original version; task 2 = Filling Task, parallel version; task 3 = Movement Task, original version; task 4 = Movement Task parallel version. Total = mean proportion. Error bars reflect the 95% confidence intervals for the mean difference of the dichotomous tasks and their score.

Fig 9 compares the conceptually congruent and the less conceptually congruent condition by showing the proportions of solved tasks for each task. It shows that the values of the conceptually congruent condition consistently lie above the values of the less conceptually congruent condition (mean difference = 11%, range = 8–14%).

Because the five-level score (0–4) cannot be normally distributed in the population we tested the conceptual congruence effect with a nonparametric U test. The effects were confirmed for the overall score (*z* = -3.74, *p* < .001) and separately for the scores of the Filling Task (*z* = -2.59, *p* = .010) and the Movement Task (*z* = -2.47, *p* < .014). The Movement Task was solved more often than the Filling Task (*z* = -7.24, *p* < .001). The parallel versions of the tasks that always were presented after the original versions were solved more often than these, as indicated by a Wilcoxon tests for paired samples, z = -3.32, p = .01.

A second ANOVA was calculated to investigate (objective) *task duration*. The mean task duration (*M* = 36.35s, *SD* = 23.02s) was used as the dependent variable. As before, *conceptual congruence* and *task direction* were used as factors. In line with previous findings (see *Introduction section*) we did not find any effect with regard to *task duration*.

The independent variables *conceptual congruence* F(1,399) = 0.18, partial η^2^ < .001, *p* = .672 and *task direction* F(1,399) = 0.03, partial η^2^ < .001, *p* = .868 as well as their interaction term F(1,399) = 0.12, partial η^2^ < .001, *p* = .727 did not reach significance.

**Fig 10:**
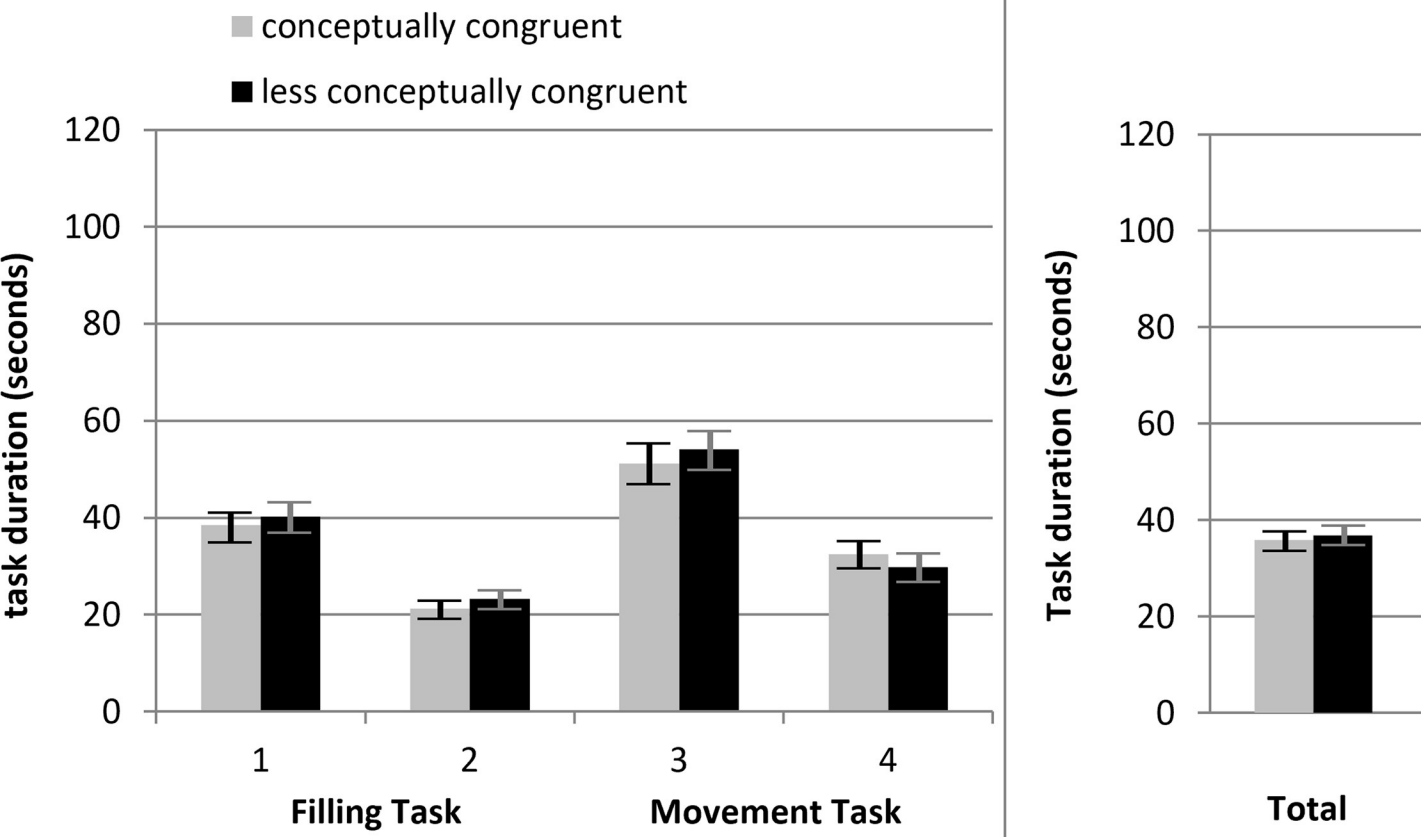
Mean task durations in seconds for each condition. Task 1 = Filling Task, original version; task 2 = Filling Task, parallel version; task 3 = Movement Task, original version; task 4 = Movement Task parallel version. Total = mean task duration. Error bars reflect the 95% confidence intervals for each mean difference.

Using t-tests for paired samples the Filling Task had a lower task duration than the Movement Task, t(402) = -7.66, Cohen’s *d* = 0.38, *p* < .001, with a mean difference of 11.1 seconds. Additionally, the original version had a higher task duration than the parallel version, t(402) = 15.11, Cohen’s *d* = 0.75, *p* < .001, with a mean difference of 19.31 seconds. Fig 10 shows the task durations for the conceptually congruent and the less conceptually congruent condition.

**Fig 11:**
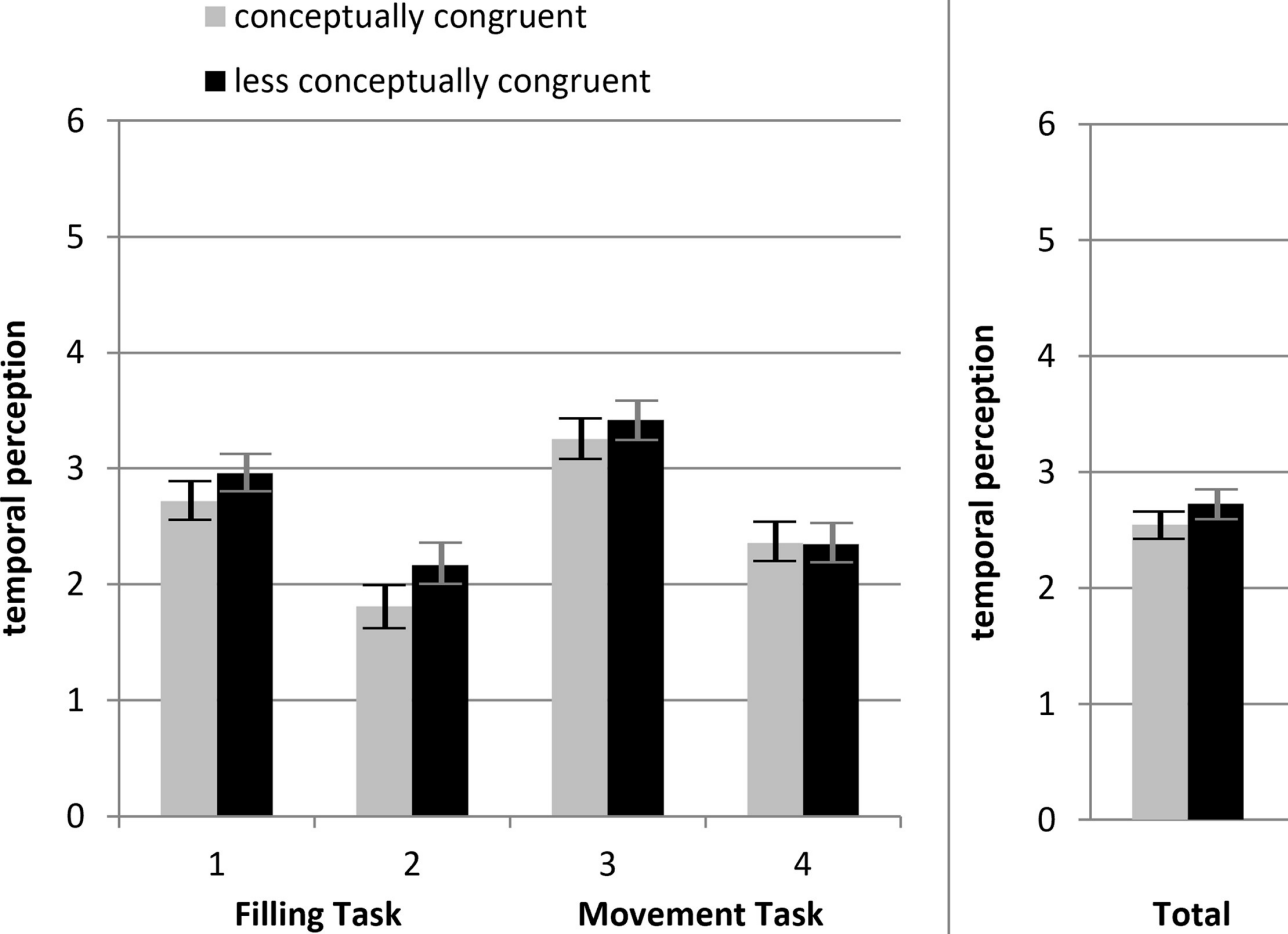
Mean temporal perception ratings for each condition. Y-axis simultaneously represents visual analogue scale and seven-point Likert scale (range: 0–6). Error bars reflect the 95% confidence intervals for each mean difference.

A third ANOVA was calculated to investigate subjective *temporal perception*. As described above temporal perception was either rated by using a seven-point Likert scale or a visual analogue scale. For comparison purposes the value ranges were equalized via linear transformation. The mean temporal perception ratings were used as the dependent variable. As before, *conceptual congruence* and *task direction* were used as factors. Additionally, *response format* was integrated as a third factor. Hypothesis 2 could not be supported. *Conceptual congruence* did not show an overall effect, F(1,386) = 2.77, partial η^2^ = .007, *p* = .097. The other independent variables *task direction* F(1,386) = 0.03, partial η^2^ < .001, *p* = .867 and *response format* F(1,386) = 067, partial η^2^ = .002, *p* = .415 also did not reach significance. The same applies for all possible two- or three-way interactions. Fig 11 shows the temporal perception ratings for the conceptually congruent and the less conceptually congruent condition.

#### Discussion

In this study, we tested Alter and Oppenheimer’s [4] assumed generalizability of fluency effects (Fig 1) in *inductive reasoning* tasks. Alter and Oppenheimer derived their model by combining several fluency findings. Results from the current study partly provide empirical evidence for the extension of the proposed processes to the field of inductive reasoning. Conceptual manipulation works in the context of inductive reasoning tasks by improving *ease of processing* (H1). However, we did not find a relationship between a conceptual manipulation and *temporal perception* (H2) or (objective) task duration. This implies that our manipulation did not work in terms of changing the perception of time. However, it did result in more correct responses in the same time (task duration). Therefore, we conclude that the manipulation led to more fluent processing.

### Study 2

In Study 1 we assumed that the variation of the symbols does not substantially change the cognitive process but only facilitates or complicates it because of more or less conceptual hints (Fig 2). The differentiation between a Filling Task and a Movement Task is based on this assumption. Theoretically, however, it is also possible to categorize the task by the implemented symbol, cross or person, and not the logic that is needed to solve the task. Therefore, we conducted a second study to examine whether our categorization into Filling Task and Movement Task was in line with the way participants actually solved the tasks.

Participants were shown the same tasks as described in Study 1 but were not asked to explicitly solve the tasks. Instead they were asked to describe the changes within the matrices in a text box. This study was important to check if the participants solve the tasks the way we thought independently of their condition, that is, independently of the symbol inside the cells being a cross or a person.

#### Sample Study 2

In total, 95 participants clicked on the recruitment link and saw the first page. In the same way as described above (see *sample 1 section*) the sample in Study 2 was reduced to a subsample of *n* = 62 participants that were included in the analysis. In this final sample the participants’ reported age ranged from 18 to 52 years (*M* = 24.1, *SD* = 7.0). The sample reportedly consisted of 60 female participants (97%) and two male participants (3%).

#### Analysis

Two independent raters judged the verbs used in the first Filling Task and the first Movement Task describing the change within the matrices. They categorized the written content into three classes: (1) content describing any kind of movement; (2) content describing a filling, adding or connecting procedure; (3) other content or content that could not clearly be assigned to Class 1 or 2. After that we calculated the accordance of the ratings and used Fisher’s exact tests and McNemar tests to examine our categorization in the Filling Task and the Movement Task.

#### Results

We calculated a Cohen’s *κ* = .83 for the accordance of the independent raters, which represents an “almost perfect” accordance [32].

On average, 95% of the descriptions of the Filling Task were rated as 2 (filling procedure), 1% were rated as 1 (movement), and 4% were rated as 3 (other). 84% of the descriptions of the Movement Task were rated as 1 (movement), 2% as 2 (filling procedure), and 14% as 3 (other). Within the Filling Task (*p* = .410) and the Movement Task (*p* = .429) there were no significant differences regarding the respective word categories between the conditions using Fisher’s exact tests.

When comparing the tasks and conditions, there were 82% correct category changes in the conceptually congruent condition and 75% correct category changes in the less conceptually congruent condition, indicating a shift from using filling as a solution strategy to movement (overall 79%). These proportions were both significant using McNemar tests (*p* < .001; *p* < .001).

#### Discussion

The results of Study 2 confirmed our assumption that for the materials used in Study 1 the change within the matrices rather than between the used symbols (crosses and persons) primarily triggers the participants’ cognitive processes. The categorization into Filling Task and Movement Task resembles the proposed solving strategies of participants working on the task closely, independent of the symbol in the cells.

## General discussion

In our studies, we demonstrated that a conceptual manipulation works in the context of inductive reasoning tasks by improving ease of processing as measured by correct responses (H1). This finding cannot be explained by a change in task duration. We conclude that the manipulation led to more fluent processing, similar to the findings by Reder [7]. We showed that the switch of symbols (crosses and persons) that are more or less conceptually congruent with the solutions of the tasks influences the rate of correct responses. The proportion of solved tasks of the conceptually congruent condition were consistently higher than the respective values of the less conceptually congruent condition (mean difference = 11%, range = 8–14%). Moreover, the variable *task direction* that could question this result was not significant. In other words, we successfully manipulated conceptual fluency (i.e., the ease of high-level operations concerned primarily with categorization and processing of a stimulus’ relation to semantic knowledge structures [9]). This is the first time that a study shows conceptual fluency effects in inductive reasoning.

However, our second hypothesis (H2), the influence on temporal perception shown by Ono and Kawahara [10], could not be supported. Even if the mean temporal perception was in fact higher in the less conceptually congruent condition, the effect only showed a trend in the predicted direction (*p* = .097). A possible explanation lies in the study by Ono and Kawahara [10], which first reported the effect. They used another operationalization of temporal perception than we did. Participants in their study had to estimate a 2,500 ms interval by clicking mouse buttons. Possibly this effect does not generalize to our operationalization of using temporal perception ratings. Perhaps more cognition is involved in the rating procedure we implemented. This could have reduced the fluency effect. Naturally, it is also conceivable that this additional effect simply does not exist in the context of inductive reasoning tasks.

### Ease of processing, fluency experience and judgments

Our mixed results illustrate the advantages of the separation of *ease of processing*, *fluency experience* and *judgments* (Fig 1). In traditional fluency studies differences in judgments are explained by differences regarding the fluency experience. This fluency experience is measured only implicitly. In our study we did not find a significant difference in judgment but we did find an effect in ease of processing, that is, more fluent processing could be observed in the conceptually congruent condition. For this reason, it is unclear if the participants in the conceptually congruent condition did not have a more fluent experience (even if they objectively processed more fluent) or if they had a more fluent experience that did not influence their temporal perception.

Forster et al. [6] used an alternative approach. They directly asked participants for their *felt fluency*. This is a convincing solution whenever measuring fluency experience is the main aim of the study. However, when investigating effects on judgments it should be a concern that responding to such an item may trigger an additional metacognitive process that could lead to possible bias. Further studies should add a *felt fluency item* as an additional experimental factor to check its metacognitive effect. Because we found evidence for fluency effects in the context of inductive reasoning, there are now some options to get deeper insights when systematically varying the demands and the presentation of the tasks in future studies (Fig 2). More research in this area will extend existing fluency models to inductive reasoning.

In future research it would be interesting to conduct a study that combines the two studies presented in this article. Such a study would enable a check for proportion solved given the conceptual condition was appropriate. That would generate more detailed information how participants think and decide. This aspect should also be included as an additional experimental factor because the request to write down own thoughts (analogue to study 2) likely influences the solution probabilities. In addition it would be interesting to implement a neutral condition by using symbols that are not congruent to neither the Filling Task nor the Movement Task (e.g. points, apples, etc.).

### Limitations of our study

We already mentioned the lack of a subjective fluency item as a possible manipulation check. Because fluent processing is fragile and a conceptual manipulation has never been transferred to inductive reasoning tasks, we focused on ensuring comparable procedures in the conceptually congruent and less conceptually congruent conditions. For this reason, we did not counterbalance the order of the Filling Task and the Movement Task and the order of the original and the parallel version. When comparing both tasks, proportion of solved tasks and task duration could be influenced by an order effect. For example, it is not certain that the Movement Task is in fact easier than the Filling Task. Differences could be explained by a learning effect of the General Inductive Reasoning Task Type. The same applies in regard to the original and the parallel version where a learning effect is highly probable. A further limitation is the somewhat unusual recruitment strategy via student assistants. Even though the sample is more diverse than a typical student sample, it is not a random sample. Therefore, the generalizability of the results is limited. Finally, we did not prevent online participants from checking their phones/watches when rating their temporal perception. However, the experimental design prevents that such behavior could have a systematic effect.

### Conclusion

This is the first study that found a conceptual fluency effect in the context of inductive reasoning. The conceptual manipulation led to more fluent processing. However, the manipulation did not influence the temporal perception ratings as expected. These mixed results illustrate that fluency effects from one area of research cannot be directly generalized to other contexts.
